# Supplementary material for: Citrus Huanglongbing is a pathogen-triggered immune disease that can be mitigated with antioxidants and gibberellin
Source: Nat Commun. 2022 Jan 26;13:529. doi: 10.1038/s41467-022-28189-9 (PMC8791970; doi:10.1038/s41467-022-28189-9)
Supplement: Supplementary file 1 — Supplementary Information [file 41467_2022_28189_MOESM1_ESM.pdf]

Table S1: Overexpression of CLas proteins containing Sec-section signals and other predicated virulence factors in *Arabidopsis*, *Citrus* and *Nicotiana*

			Overexpressed in *Arabidopsis thaliana*			Overexpressed in *Citrus*			Overexpressed in *N. tabacum*			Reference
Locus	Annotation	SDE	PCR	WB	Phenotype	PCR	WB	Phenotype	PCR	WB	Phenotype	
CLIBASIA_00460		Y	+	+	Normal							
CLIBASIA_00530		Y	+	+	Normal							
CLIBASIA_02145		Y	+	-	Normal							
CLIBASIA_02215		Y	+	+	Normal							
CLIBASIA_02470		Y	+	-	Normal							
CLIBASIA_02935		Y	+	-	Normal				+	-	Normal	
CLIBASIA_03695		Y	+	+	Normal							
CLIBASIA_03975		Y	+	-	Normal							
CLIBASIA_04320		Y	+	-	Normal							
CLIBASIA_04580		Y	+	-	Normal							
CLIBASIA_04735		Y	+	-	Normal							
CLIBASIA_05115	predicated virulence factor	Y	+	+	Normal							
CLIBASIA_05320		Y	+	-	Normal							
CLIBASIA_05330		Y	+	+	Normal							
CLIBASIA_04260	OmpA		+	+	Normal							
CLIBASIA_03315	predicated virulence factor		+	+	Normal							
CLIBASIA_03105	Flp/Fap pilin component		+	+	Normal							
CLIBASIA_05640		Y	+	+	Normal							
CLIBASIA_02305		Y	+	+	Normal							
CLIBASIA_03085		Y	+	+	Normal							
CLIBASIA_00420	outer membrane lipoprotein		+	+	Normal							
CLIBASIA_01300	predicated virulence factor		+	-	Normal							
CLIBASIA_01345	serralysin		+	-	Normal				+	-	Normal	

CLIBASIA_02425	outer membrane protein		+	-	Normal							
CLIBASIA_03295	predicated virulence factor		+	-	Normal							
CLIBASIA_04055	predicated virulence factor		+	+	Normal							
CLIBASIA_04520	predicated virulence factor		+	-	Normal				+	-	Normal	
CLIBASIA_04410		Y	+	+	Normal							
CLIBASIA_05150		Y	+	+	Normal							
CLIBASIA_01640	predicated virulence factor		+	+	Normal							
CLIBASIA_04865	predicated virulence factor		+	+	Normal							
CLIBASIA_04405		Y	+	+	Normal	+	+	Normal	+	+	Normal	
CLIBASIA_05160	predicated virulence factor		+	+	Normal							
CLIBASIA_05475	predicated virulence factor		+	+	Normal							
CLIBASIA_04025	SDE15	Y	+	+	Normal	+	+	Normal	+	+	Normal	[1]
CLIBASIA_05315	SDE1	Y				+	+	Normal	+	+	Normal	[2]
CLIBASIA_02845		Y				+	+	Normal	+	+	Normal	
CLIBASIA_00255	SahA								+	+	Normal	[3]
CLIBASIA_00830	cytochrome-c oxidase								+	-	Normal	
CLIBASIA_04330		Y							+	+	Normal	
CLIBASIA_00470		Y							+	+	Normal	
CLIBASIA_02160	metalloprotease								+	+	Normal	
CLIBASIA_04030		Y							+	+	Normal	
CLIBASIA_00520		Y							+	-	Normal	
CLIBASIA_02395	TPR repeat-containing protein								+	-	Normal	
CLIBASIA_04040		Y							+	-	Normal	
CLIBASIA_01555	hemolysin								+	+	Normal	

Note: Normal indicates that the phenotypes of transgenic plants are same as wild type plants. WB: Western blot. SDE: Sec-dependent effector. Y: indicates presence of Sec-secretion signal. "+" indicates presence. "-" indicates absence.

Table S2: Transcriptomic studies of sweet orange in response to CLas infection that were used for GO enrichment analysis in this study

Study	Genotype	Number of samples	Tissue	Time after CLas infection	Repository number	Sequencing method	Analysis method	Analysis statistic	Condition	Reference
1	Valencia	6 (3 healthy + 3 HLB)	Stem	16 months	GSE33004	Microarray	Limma R Package	Unadjusted $P < 0.05$ FC > 2	greenhouse	Aritua et al., 2013 [4]
2	Valencia	5 (2 healthy + 3 HLB)	Leaf	5-9 weeks	GSE33459	Microarray	Limma R Package	Unadjusted $P < 0.05$ FC > 2	greenhouse	Albrecht et al., 2008 [5]
3	Valencia	5 (2 healthy + 3 HLB)	Leaf	13-17 weeks	GSE33459	Microarray	Limma R Package	Unadjusted $P < 0.05$ FC > 2	greenhouse	Albrecht et al., 2008 [5]
4	Sweet orange	6 (3 healthy + 3 HLB)	Leaf	8 months	GSE33003	Microarray	Limma R Package	Unadjusted $P < 0.05$ FC > 2	greenhouse	Kim et al., 2009 [6]
5	Madam Vinous	6 (3 healthy + 3 HLB)	Leaf	7 months	GSE29633	Microarray	Limma R Package	Unadjusted $P < 0.05$ FC > 2	greenhouse	Fan et al., 2011 [7]
6	Madam Vinous	6 (3 healthy + 3 HLB)	Leaf	5 weeks	Not available. Data within the manuscript.	Microarray	Limma R Package	Unadjusted $P < 0.05$ FC > 2	greenhouse	Fan et al., 2012 [8]
7	Madam Vinous	6 (3 healthy + 3 HLB)	Leaf	17 weeks	Not available. Data within the manuscript.	Microarray	Limma R Package	Unadjusted $P < 0.05$ FC > 2	greenhouse	Fan et al., 2012 [8]
8	Madam Vinous	6 (3 healthy + 3 HLB)	Leaf	27 weeks	Not available. Data within the manuscript.	Microarray	Limma R Package	Unadjusted $P < 0.05$ FC > 2	greenhouse	Fan et al., 2012 [8]
9	Hamlin	8 (4 healthy + 4 HLB)	juice vesicles	_	GSE33373	Microarray	Limma R Package	Unadjusted $P < 0.05$ FC > 2	Field	Liao et al., 2012 [9]
10	Hamlin	8 (4 healthy + 4 HLB)	vascular tissue	_	GSE33373	Microarray	Limma R Package	Unadjusted $P < 0.05$ FC > 2	Field	Liao et al., 2012 [9]
11	Hamlin	8 (4 healthy + 4 HLB)	flavedo	_	GSE33373	Microarray	Limma R Package	Unadjusted $P < 0.05$ FC > 2	Field	Liao et al., 2012 [9]
12	Hamlin	8 (4 healthy + 4 HLB)	Seed	_	GSE33373	Microarray	Limma R Package	Unadjusted $P < 0.05$ FC > 2	Field	Liao et al., 2012 [9]
13	Valencia	8 (4 healthy + 4 HLB)	juice vesicles	_	GSE33373	Microarray	Limma R Package	Unadjusted $P < 0.05$ FC > 2	Field	Liao et al., 2012 [9]
14	Valencia	8 (4 healthy + 4 HLB)	vascular tissue	_	GSE33373	Microarray	Limma R Package	Unadjusted $P < 0.05$ FC > 2	Field	Liao et al., 2012 [9]
15	Valencia	8 (4 healthy + 4 HLB)	flavedo	_	GSE33373	Microarray	Limma R Package	Unadjusted $P < 0.05$ FC > 2	Field	Liao et al., 2012 [9]
16	Valencia	10 (5 healthy + 5 HLB), biologic replicates merged as one sample for each group	Immature fruit	_	SRP022979	RNA-seq	DESeq	Adjusted $P < 0.05$ FC > 2	Field	Martinelli et al., 2013 [10]

17	Valencia	10 (5 healthy + 5 HLB), biologic replicates merged as one sample for each group	Mature fruit	_	SRP022979	RNA-seq	DESeq	Adjusted $P < 0.05$ FC > 2	Field	Martinelli et al., 2013 [10]
18	Hamlin	4(2 healthy+2 HLB)	Dropped fruit	_	GSE101381	RNA-seq	DESeq2	Adjusted $P < 0.05$ FC > 2	Field	Zhao et al., 2019 [11]
19	Hamlin	4(2 healthy+2 HLB)	Retained fruit	_	GSE101381	RNA-seq	DESeq2	Adjusted $P < 0.05$ FC > 2	Field	Zhao et al., 2019 [11]
20	Madam Vinous	6 (3 healthy + 3 HLB)	Leaf	7 weeks	Not available. Data within the manuscript.	RNA-seq	DESeq	Adjusted $P < 0.05$ FC > 2	greenhouse	Yu et al., 2017 [12]

Table S3: GO enrichment analysis of DEGs of *Citrus sinensis* in response to CLas infection based on nine different studies as specified in Table S2

GO_acc	Term type	Term	Query item	Bg item	p-value	FDR
GO:0044464	C	cell part	880	1618	1.60E-49	3.20E-47
GO:0005623	C	cell	880	1618	1.60E-49	3.20E-47
GO:0044262	P	cellular carbohydrate metabolic process	92	111	2.70E-12	6.00E-09
GO:0005506	F	iron ion binding	185	342	2.40E-10	3.70E-07
GO:0003700	F	transcription factor activity	174	342	2.40E-08	1.80E-05
GO:0003677	F	DNA binding	351	852	4.20E-07	0.00016
GO:0003824	F	catalytic activity	2022	6096	4.20E-07	0.00016
GO:0016491	F	oxidoreductase activity	492	1277	1.20E-06	0.00036
GO:0043169	F	cation binding	529	1398	2.50E-06	0.00062
GO:0046872	F	metal ion binding	524	1392	4.40E-06	0.00095
GO:0008152	P	metabolic process	2064	6289	4.00E-06	0.0029
GO:0009987	P	cellular process	1587	4729	3.80E-06	0.0029
GO:0055114	P	oxidation reduction	443	1155	6.20E-06	0.0034
GO:0016879	F	ligase activity, forming carbon-nitrogen bonds	37	47	1.90E-05	0.0036
GO:0043565	F	sequence-specific DNA binding	91	174	2.30E-05	0.0039
GO:0046914	F	transition metal ion binding	378	983	2.60E-05	0.004
GO:0042221	P	response to chemical stimulus	84	159	3.50E-05	0.015
GO:0020037	F	heme binding	165	396	0.00034	0.036
GO:0016705	F	oxidoreductase activity, acting on paired donors, with incorporation or reduction of molecular oxygen	144	335	0.00028	0.036
GO:0046906	F	tetrapyrrole binding	165	396	0.00034	0.036
GO:0043167	F	ion binding	529	1481	0.00029	0.036
GO:0005996	P	monosaccharide metabolic process	23	25	0.00015	0.045
GO:0019318	P	hexose metabolic process	22	23	0.00015	0.045
GO:0005976	P	polysaccharide metabolic process	47	77	0.00016	0.045

Notes: The *P* value was determined using Fisher's exact test method and corrected using the FDR method.

References and Notes

1 Pang, Z. *et al.* Citrus CsACD2 Is a Target of Candidatus Liberibacter Asiaticus in Huanglongbing Disease. *Plant Physiol* **184**, 792-805, doi:10.1104/pp.20.00348 (2020).

2 Clark, K. *et al.* An effector from the Huanglongbing-associated pathogen targets citrus proteases. *Nat Commun* **9**, 1718, doi:10.1038/s41467-018-04140-9 (2018).

3 Li, J. *et al.* '*Candidatus* Liberibacter asiaticus' Encodes a Functional Salicylic Acid (SA) Hydroxylase That Degrades SA to Suppress Plant Defenses. *Mol Plant Microbe Interact* **30**, 620-630, doi:10.1094/MPMI-12-16-0257-R (2017).

4 Aritua, V., Achor, D., Gmitter, F. G., Albrigo, G. & Wang, N. Transcriptional and Microscopic Analyses of Citrus Stem and Root Responses to *Candidatus* Liberibacter asiaticus Infection. *PLoS One* **8**, e73742, doi:10.1371/journal.pone.0073742 (2013).

5 Albrecht, U. & Bowman, K. Gene expression in Citrus sinensis (L.) Osbeck following infection with the bacterial pathogen *Candidatus* Liberibacter asiaticus causing Huanglongbing in Florida. *PLANT SCIENCE* **175**, 291-306 (2008).

6 Kim, J. S., Sagaram, U. S., Burns, J. K., Li, J. L. & Wang, N. Response of sweet orange (Citrus sinensis) to '*Candidatus* Liberibacter asiaticus' infection: microscopy and microarray analyses. *Phytopathology* **99**, 50-57, doi:10.1094/PHYTO-99-1-0050 (2009).

7 Fan, J. *et al.* Comparative iTRAQ proteome and transcriptome analyses of sweet orange infected by "Candidatus Liberibacter asiaticus". *Physiol Plant* **143**, 235-245, doi:10.1111/j.1399-3054.2011.01502.x (2011).

8 Fan, J. *et al.* Comparative transcriptional and anatomical analyses of tolerant rough lemon and susceptible sweet orange in response to '*Candidatus* Liberibacter asiaticus' infection. *Mol Plant Microbe Interact* **25**, 1396-1407, doi:10.1094/MPMI-06-12-0150-R (2012).

9 Liao, H. L. & Burns, J. K. Gene expression in Citrus sinensis fruit tissues harvested from huanglongbing-infected trees: comparison with girdled fruit. *J Exp Bot* **63**, 3307-3319, doi:10.1093/jxb/ers070 (2012).

10 Martinelli, F. *et al.* Gene regulatory networks elucidating huanglongbing disease mechanisms. *PLoS One* **8**, e74256, doi:10.1371/journal.pone.0074256 (2013).

11 Zhao, W., Baldwin, E. A., Bai, J., Plotto, A. & Irey, M. Comparative analysis of the transcriptomes of the calyx abscission zone of sweet orange insights into the huanglongbing-associated fruit abscission. *Hortic Res* **6**, 71, doi:10.1038/s41438-019-0152-4 (2019).

12 Yu, Q. *et al.* Reprogramming of a defense signaling pathway in rough lemon and sweet orange is a critical element of the early response to '*Candidatus* Liberibacter asiaticus’. *Hortic Res* **4**, 17063, doi:10.1038/hortres.2017.63 (2017).

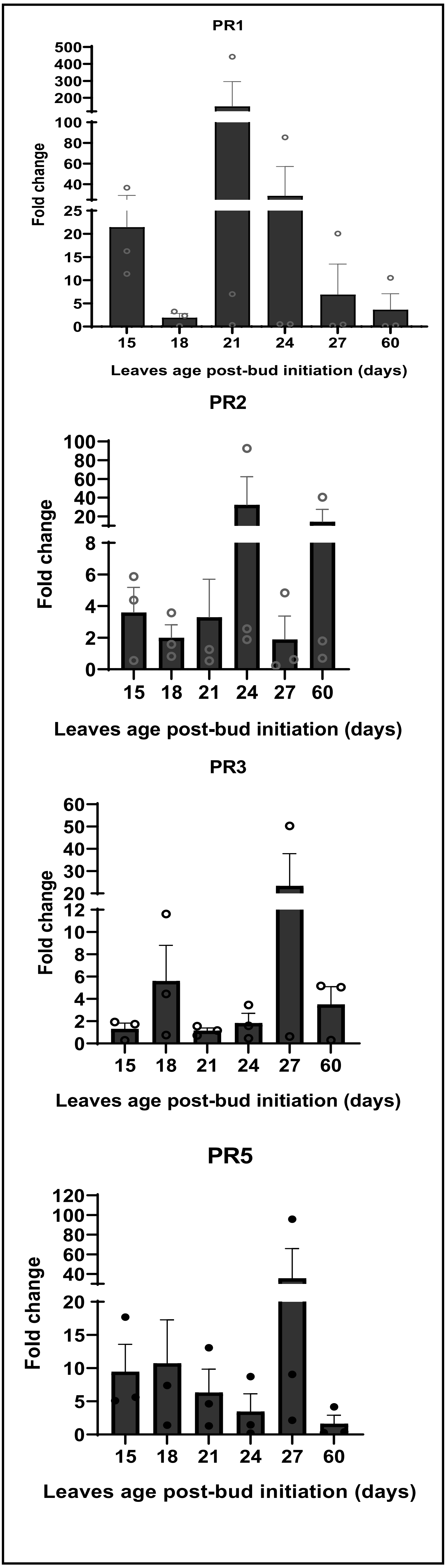

Fig. S1. Temporal expression of immune-related genes in response to CLas infection of young flush of *C. sinensis.* The reverse transcription-quantitative PCR (RT-qPCR) analysis was conducted using young leaves from three two-year-old HLB positive Valencia sweet orange trees compared with that of three healthy Valencia trees with one tree as one biological replicate. Experiments were repeated two times with similar results and representative results are shown. Data shown are mean ± SEM, n=3.

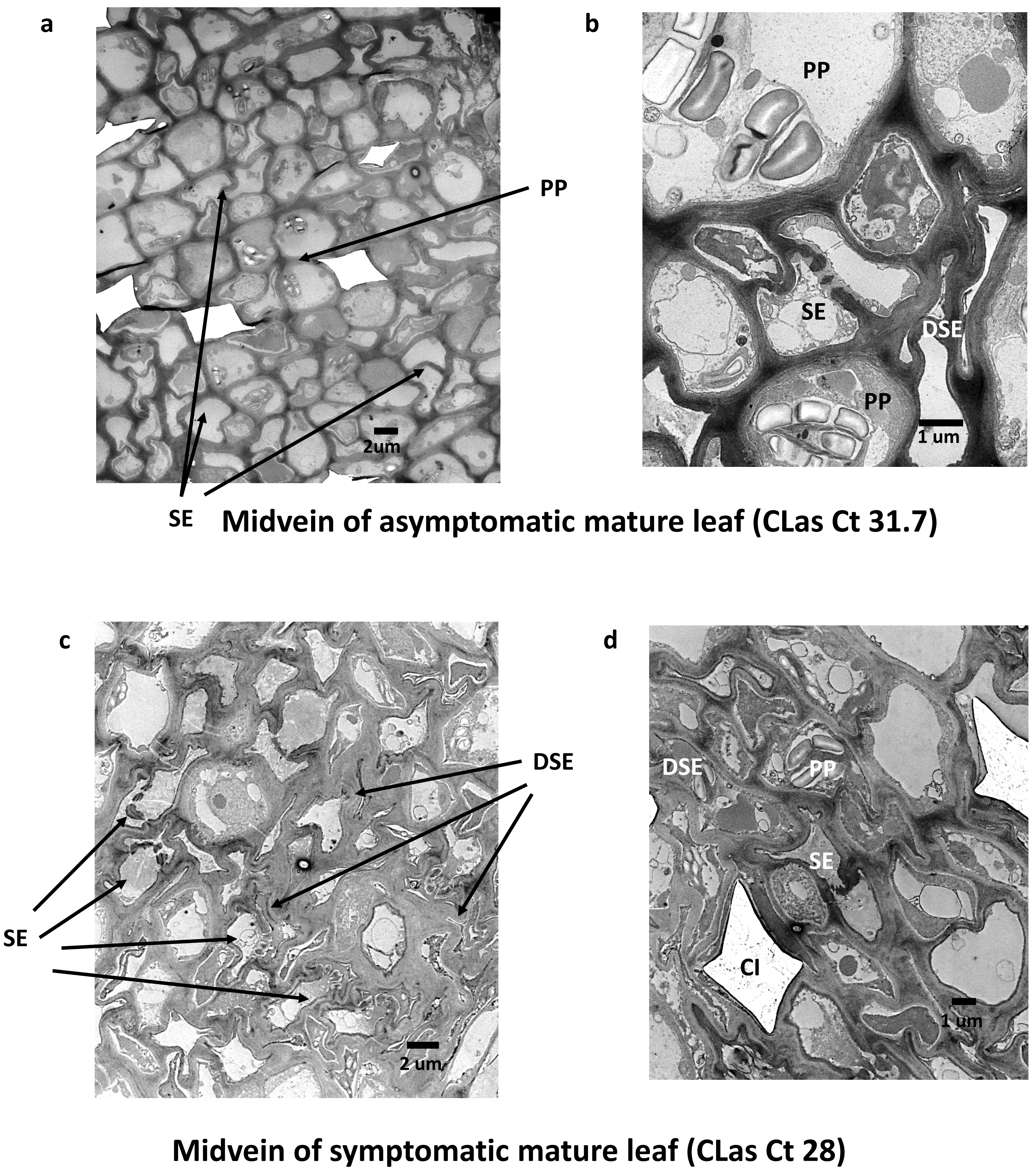

Fig. S2. TEM observation of the midribs of mature leaves of *C. sinensis* trees grown in the field that are HLB positive*.* (a and b) Midvein of asymptomatic mature leaf. (c and d) Midvein of symptomatic mature leaf. CLas titers were indicated by Ct values for the samples used. Scale bar for each picture is included. SE: sieve element. DSE: dead sieve element cells. PP: parenchyma cells. CI: calcium oxalate crystal idioblas.

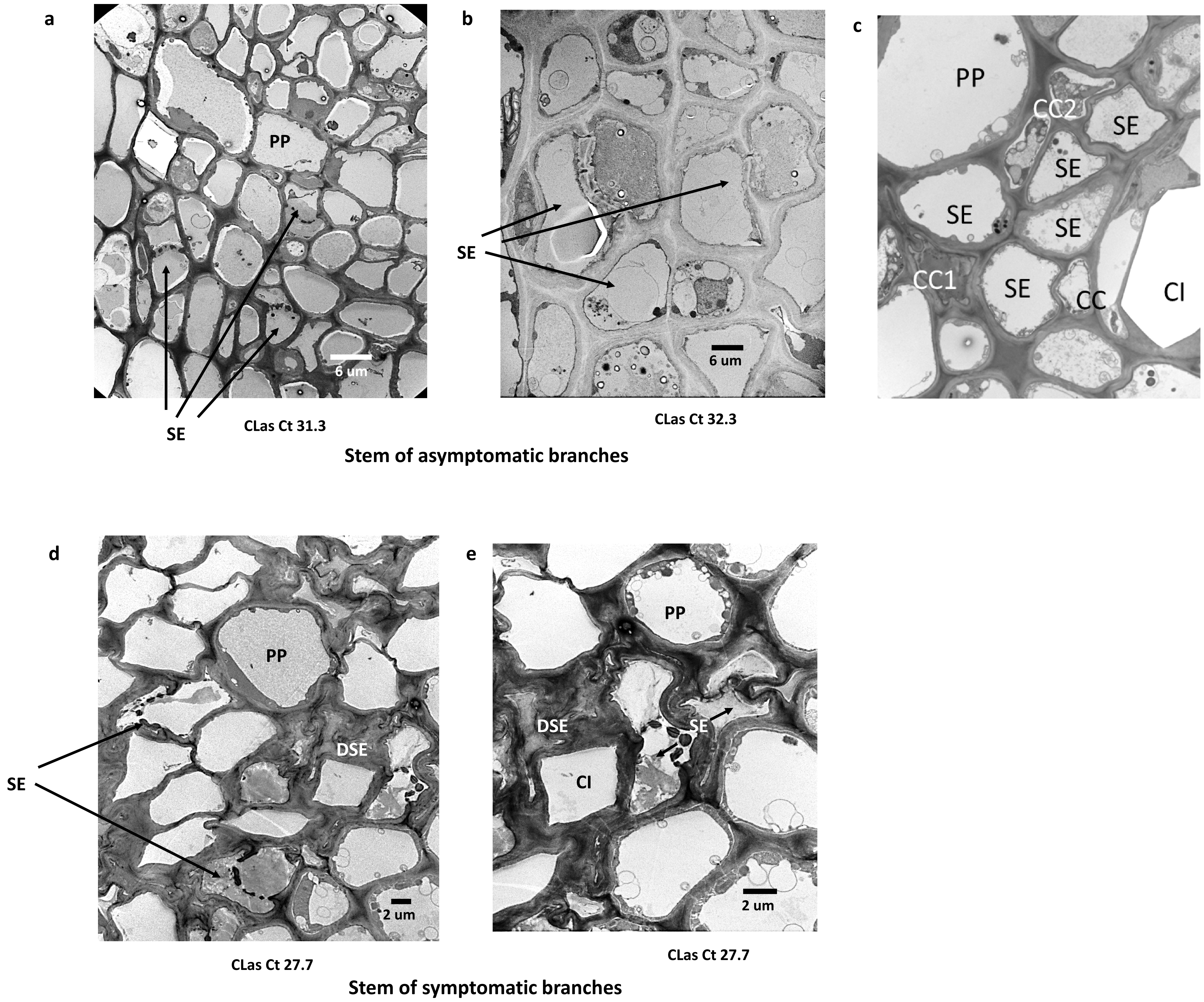

Fig. S3. TEM observation of the stem tissues of HLB positive *C. sinensis* trees grown in the field. Stems were collected from branches without HLB symptoms (a, b and c) and branches with HLB symptoms (d and e). Scale bar for each picture is included. SE: sieve element. DSE: dead sieve element cells. PP: parenchyma cells. CI: calcium oxalate crystal idioblas. CLas titers in the tested samples were indicated by Ct values.

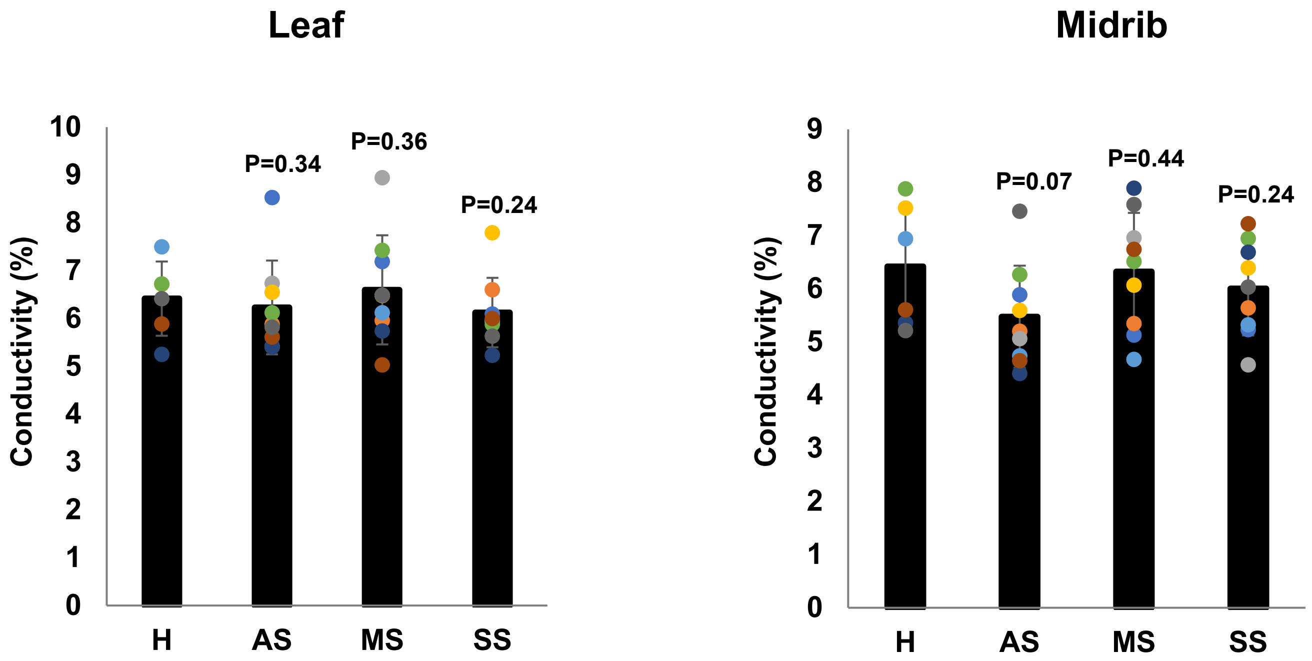

Fig. S4. Comparison of ion leakage activities of leaves of CLas-negative and CLas-infected sweet orange trees. H: healthy leaves. AS: asymptomatic leaves. MS: leaves with mild symptoms. SS: leaves with severe symptoms. Healthy leaves were collected from CLas-free sweet orange plants. AS, MS, and SS were collected from CLas positives sweet orange trees in the groves. For statistical significance tests, two tailed Student's t-test was conducted (n=9 for AS, MS and SS; n=6 for H). Mean and standard deviation are shown. P values for comparisons against CLas negative samples were shown above each column. Experiments were repeated two times with similar results and representative results are shown. Source data are provided as a Source Data file.

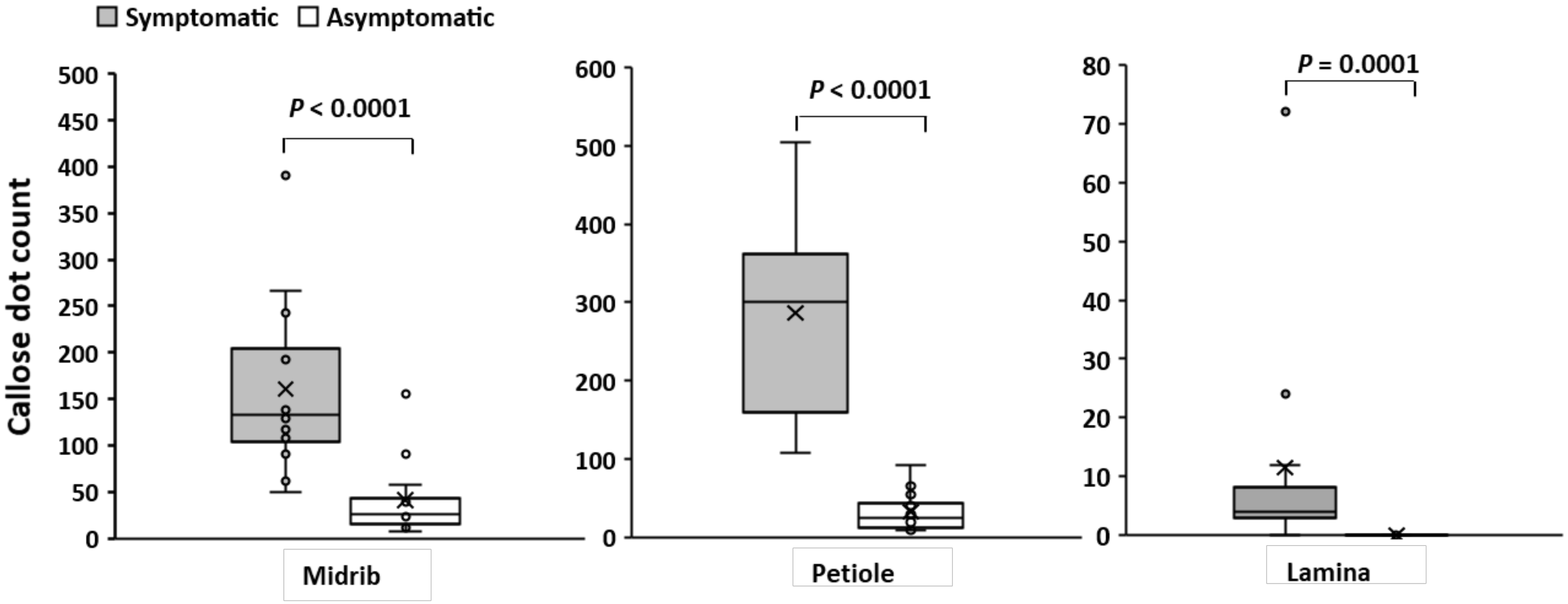

Fig. S5. CLas infection induces callose deposition in phloem tissues of *C. sinensis*. *C. sinensis* 'Hamlin' leaf samples were fixed with FAA solution overnight, sectioned and stained with 0.005% aniline blue solution prior to analysis. 12 representatives for each sample type were examined and callose dots were counted per slide area (24.75 mm^2). Statistical analysis was performed using two-tailed Student's t test based on Log(x+1) transformed data. Box plots: central line represents the median, box edges delimit lower and upper quartiles, and dots above the boxes are outliers.

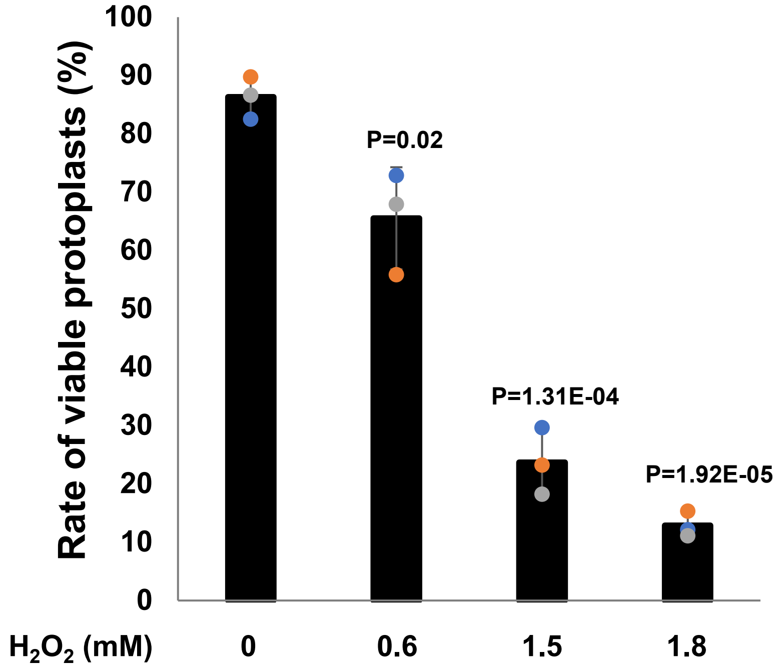

Fig. S6. H_2O_2 kills protoplast cells of *C. sinensis*. Freshly prepared protoplast cells of *C. sinensis* were treated with different concentrations of H_2O_2 for 24 h and tested for viability via fluorescein diacetate (FDA) staining. Each experiment contains three biological replicates. Mean and SD were shown. Statistical differences were analyzed using one way ANOVA with Bonferroni Correction. P values are labeled above column. Experiments were repeated two times with similar results and representative results are shown. Source data are provided as a Source Data file.

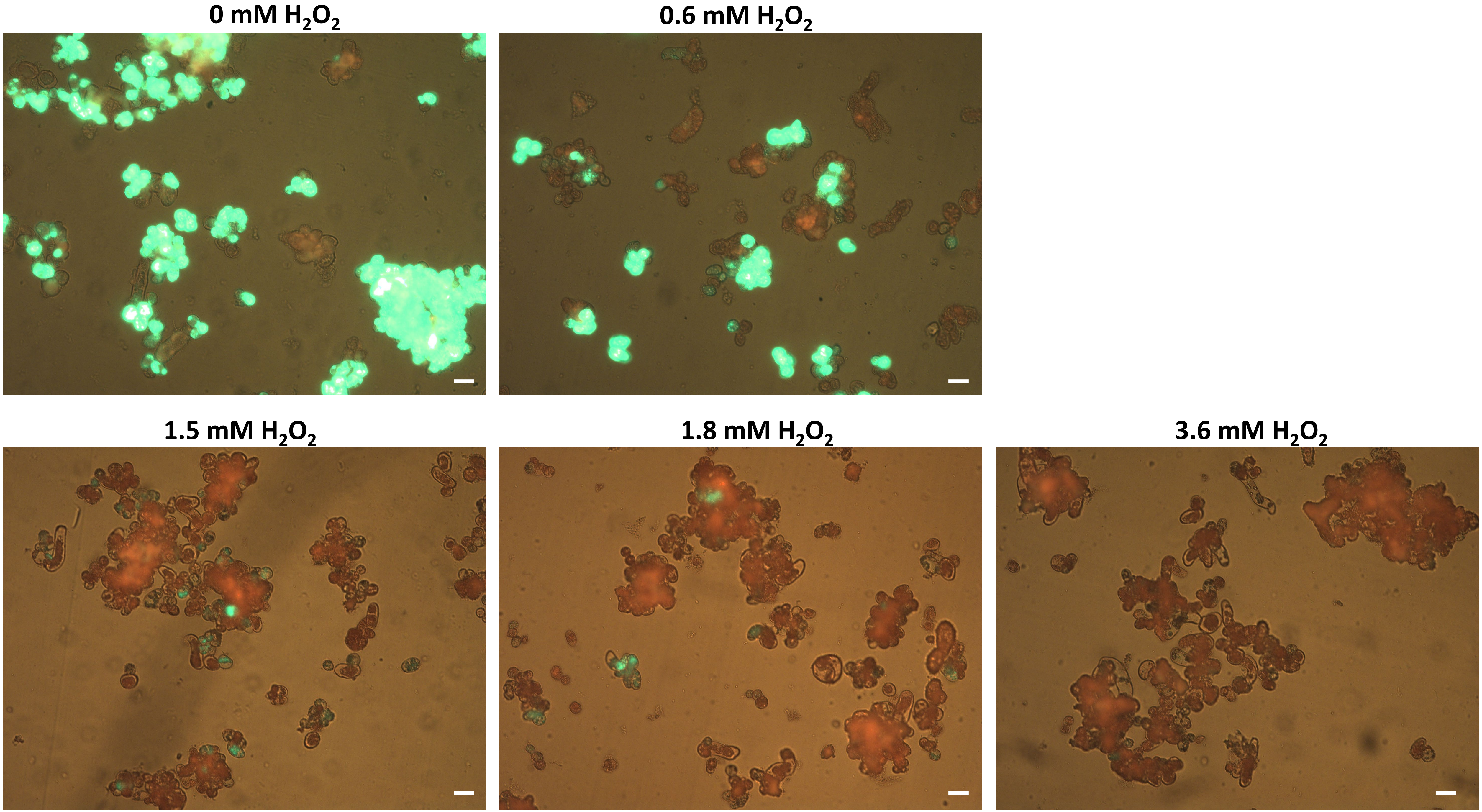

Fig. S7. Viability of *Citrus sinensis* 'Hamlin' suspension culture cells treated with H_2O_2. The suspension culture was treated with H_2O_2 for 24 hours. Each sample was stained with fluorescein diacetate (stain only living cells, green color) and propidium iodide (stain only dead cells, orange to red color). Bar size, 20 mm. Representative pictures are shown.

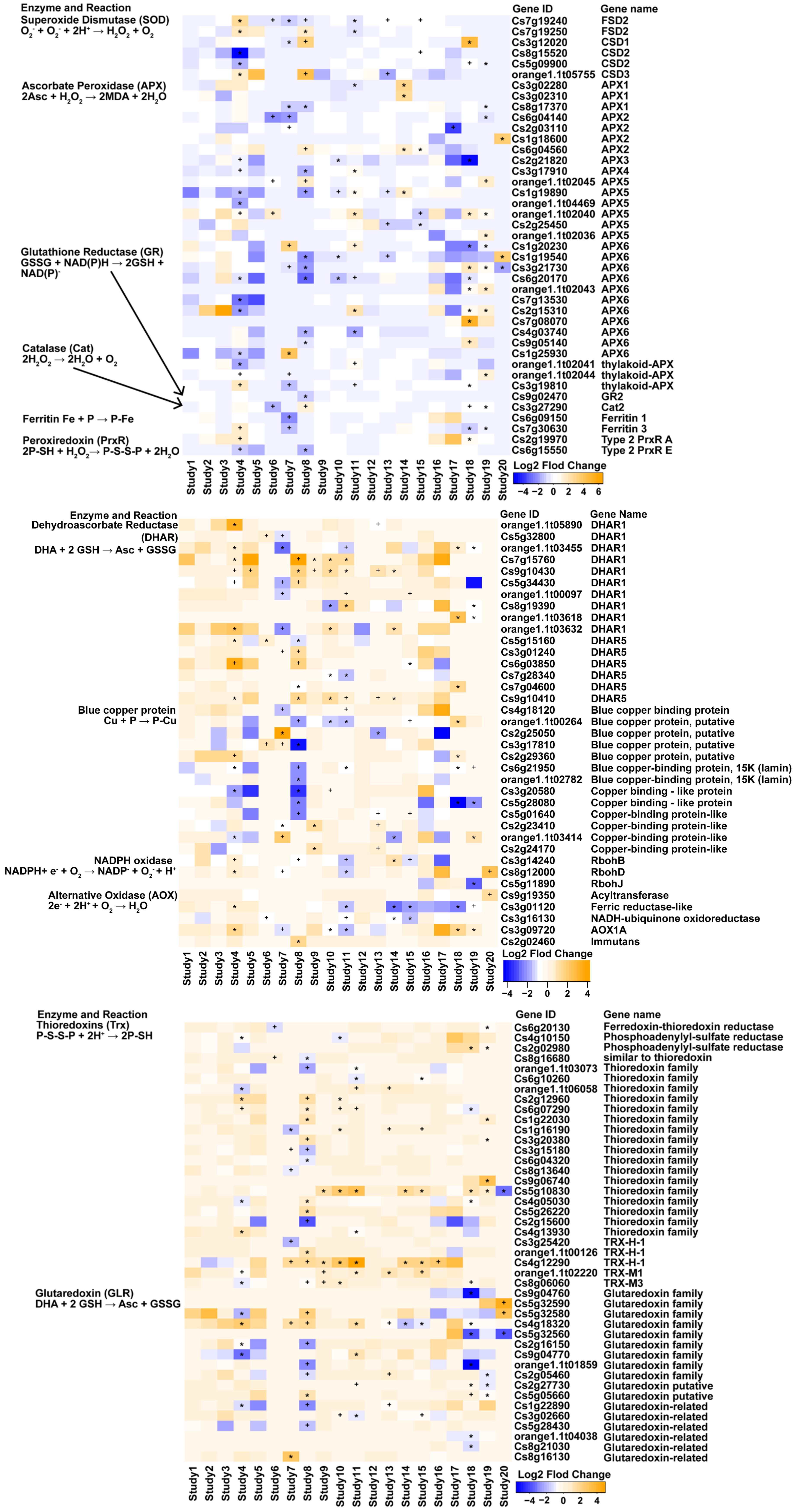

Fig. S8. The expression profile of ROS-related genes in response to CLas infection in 9 previous studies. Affiliation of each gene is indicated in *brackets*. *Orange* denotes "higher in CLas infected than CLas negative samples" while blue denotes "higher in CLas negative than CLas infected samples". The asterisk denotes *P* value < 0.01; the plus sign denotes *P* value < 0.05. The information about the 9 previous studies is listed in Table S2.

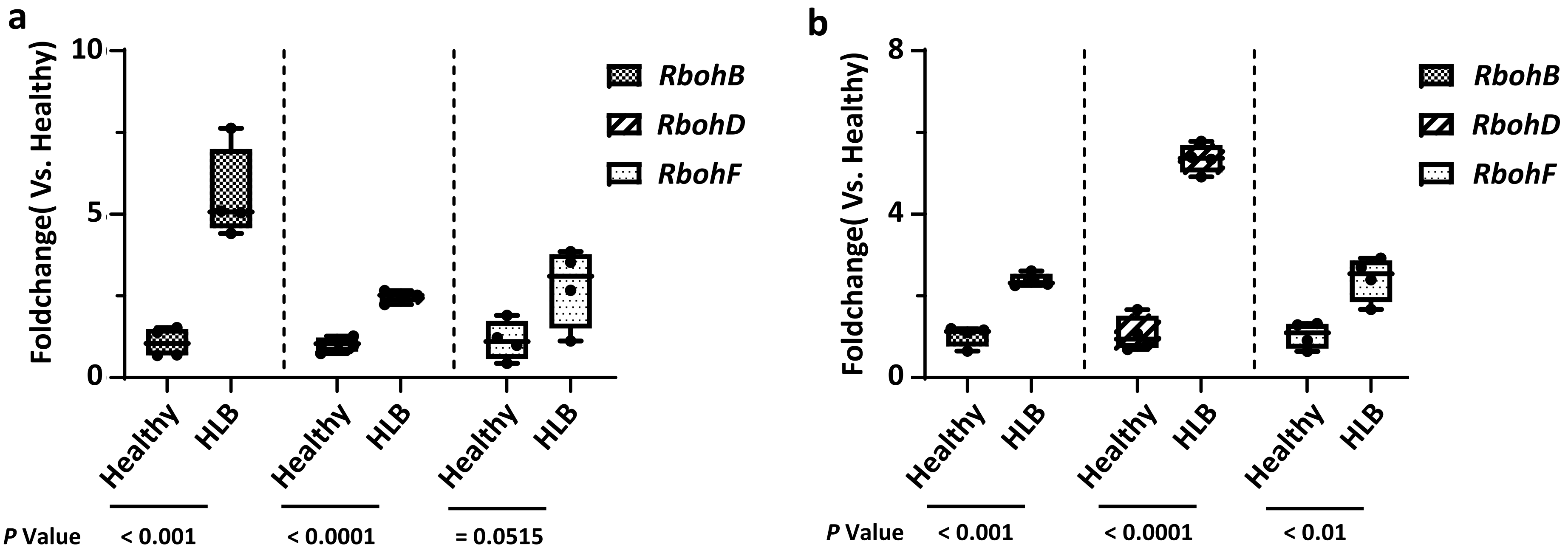

Fig. S9. Expression of NADPH oxidase genes in response to CLas infection. qRT-PCR analysis of *RBOHB, RBOHD, and RBOHF* in healthy and HLB positive sweet orange leaves. a. Samples collected from *C. sinensis* 'Valencia' trees grown in the greenhouse. b. Samples collected from *C. sinensis* 'Valencia' trees grown in the field. The housekeeping gene *GAPDH* encoding glyceraldehyde-3- phosphate dehydrogenase-C was used as an endogenous control. Box plots: central line represents the median, box edges delimit lower and upper quartiles, and dots represent the foldchange of each replicate (n=4). *P* value listed below was calculated by two-tailed Student's t-test. The experiments were repeated twice with similar results. Source data are provided as a Source Data file.

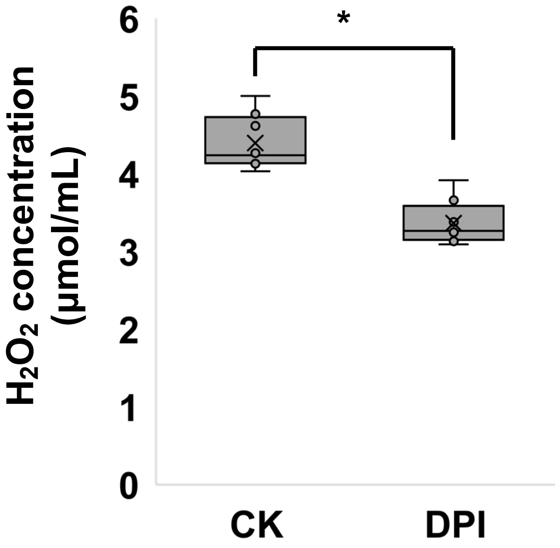

Fig. S10. ROS levels in CLas-positive stems were reduced by NADPH oxidase inhibitor diphenyleneiodonium (DPI). HLB positive branches from the summer flush of Valencia sweet orange in the field were collected and then soaked in DPI solution (25 µM) or water (control). After 48 h treatment, phloem-enriched bark tissues were collected for H_2O_2 concentration measurement. Eight biological replicates were used for this test. * indicates $p < 0.01$ (two-tailed Student's t-test). The experiment was repeated twice with similar results.

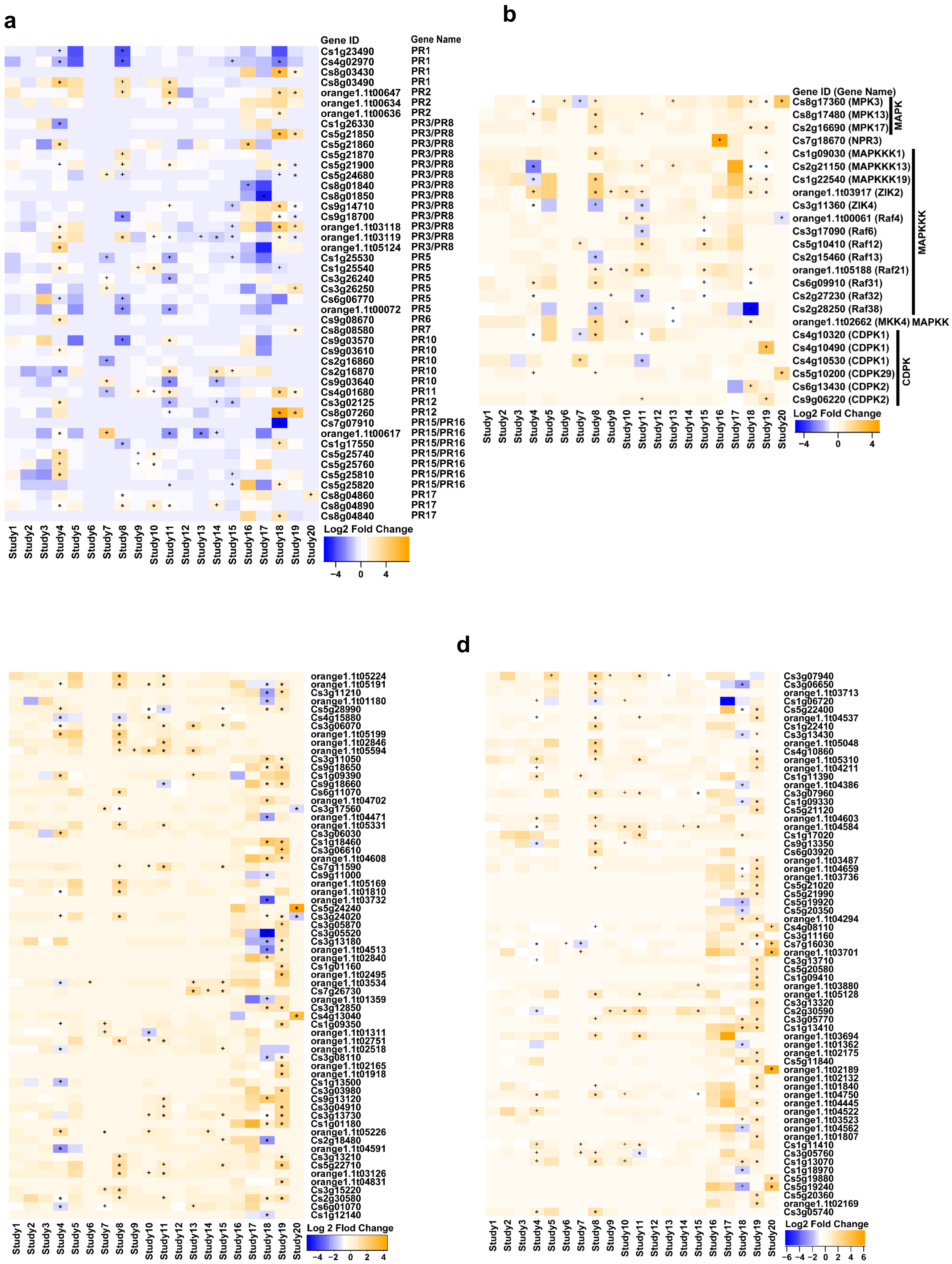

Fig. S11. The expression profile of immune related genes, including PR, MAPK and NBS-LRR genes in response to CLas infection in 9 previous studies. Affiliation of each gene is indicated in *brackets*. *Orange* denotes "higher in CLas infected than CLas negative samples" while blue denotes "higher in CLas negative than CLas infected samples". The asterisk denotes *P* value < 0.01; the plus sign denotes *P* value < 0.05. (a) PR genes, (b) MAPK genes. (c and d) NBS-LRR genes. The information about the 9 previous studies is listed in Table S2.

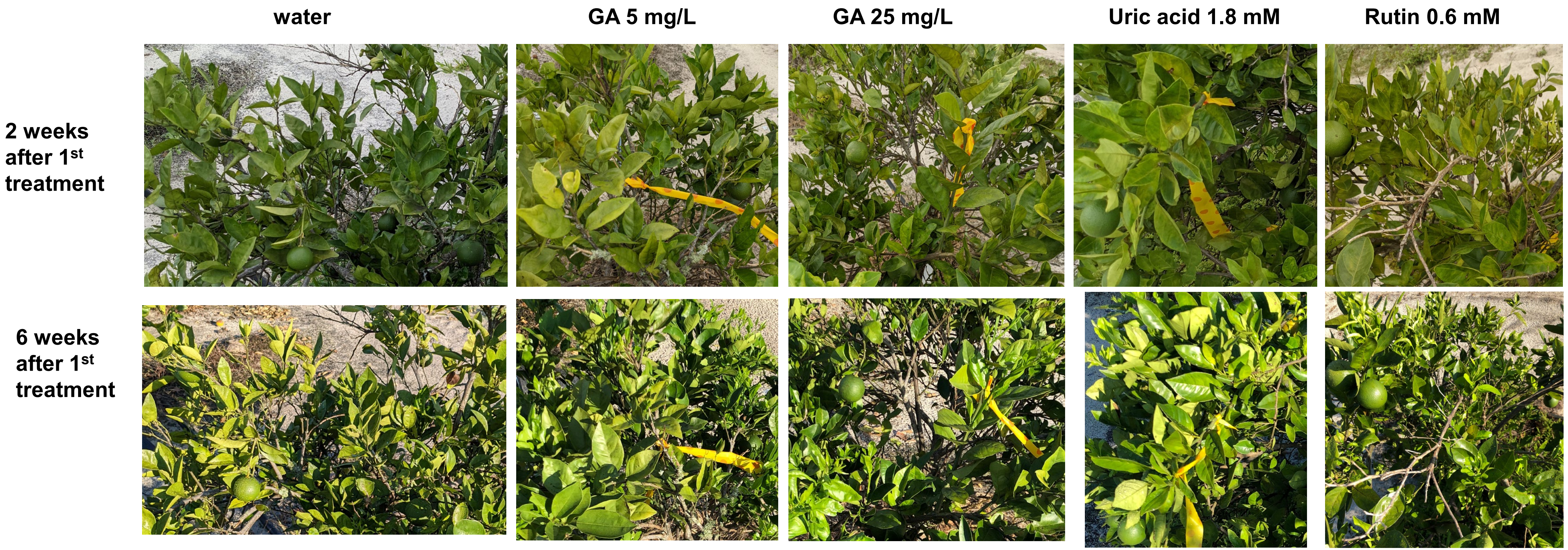

Fig. S12. Effect of GA and antioxidants treatment on HLB symptoms. To test the effect of GA treatment on HLB symptoms, HLB positive *C. sinensis* 'Valencia' trees were treated with GA and antioxidants (uric acid and rutin) via foliar spray weekly. Representative branches were selected to demonstrate symptom changes.

a

b

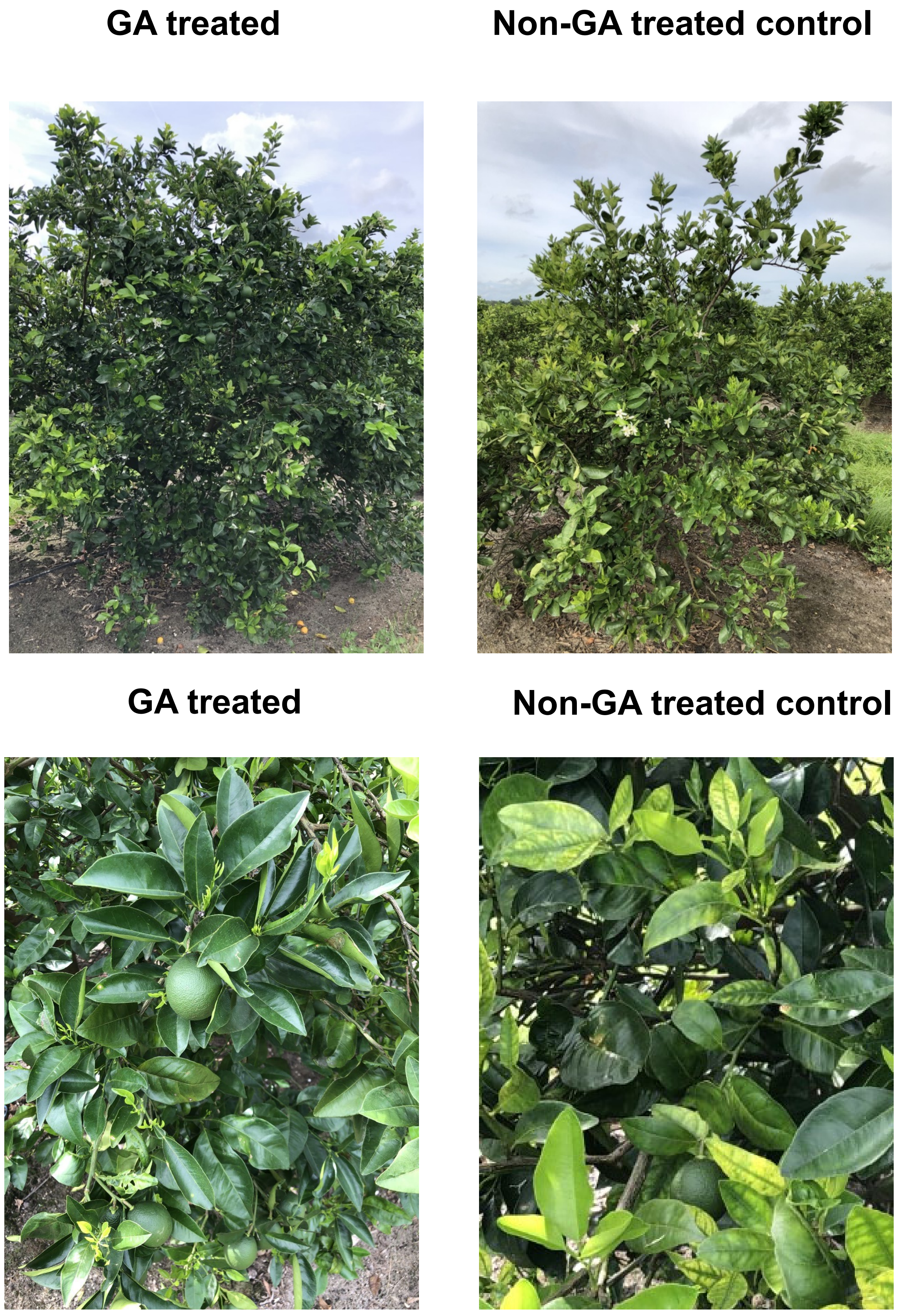

c

	GA treated	Non-GA treated control
HLB disease incidence	100%	100%
Ratio of symptomatic leaves/total leaves	0.43 ± 0.05	0.57 ± 0.03

*

Fig. S13. Gibberellin (GA) treatment of *C. sinensis* suppresses HLB. *C. sinensis* 'Vernia' blocks were treated with GA (1247 ppm) in November 2020. Nearby blocks of *C. sinensis* 'Vernia' that were not treated with GA were used as negative controls. Symptoms, HLB disease incidence and ratio of symptomatic leaves/total leaves were investigated in June 2021. (a) Representative whole trees. (b) Representative sections. (c) HLB disease incidence and ratio of symptomatic leaves vs total leaves in different treatments. Pictures were taken at the same day in June 2021. * indicates p value < 0.05 based on Student's t-test.

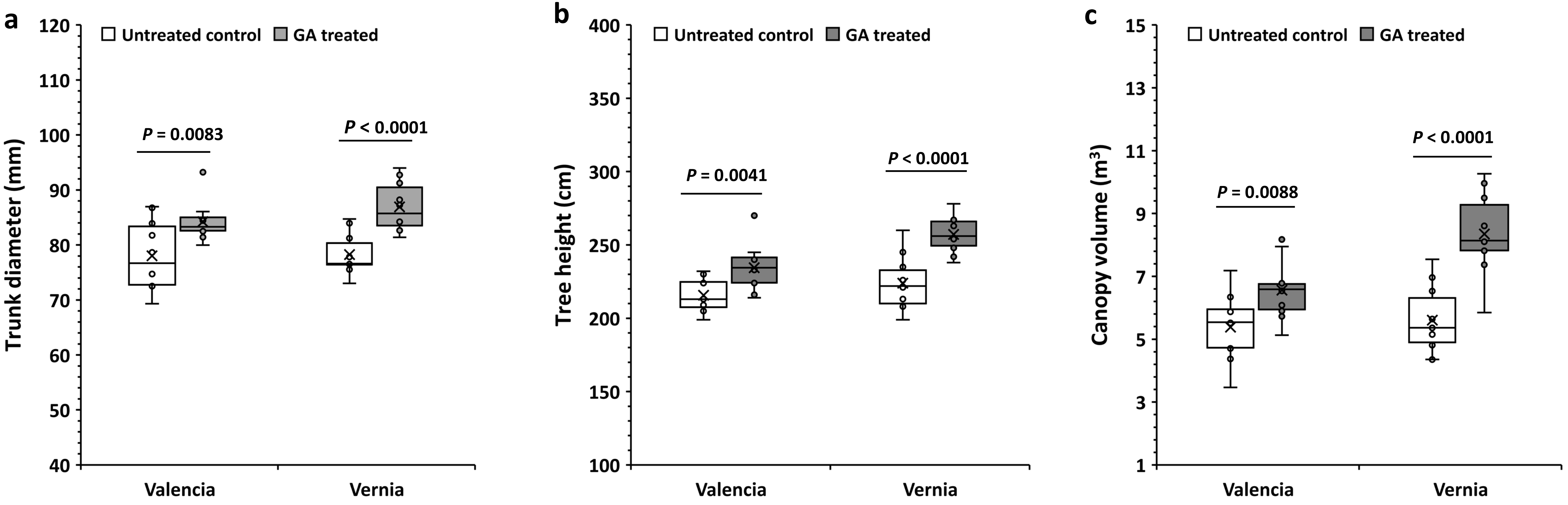

Fig. S14. **Growth performance of citrus trees (two cultivars: Valencia and Vernia) treated by GA in commercial groves in FL.** *C. sinensis* blocks were treated with GA (1247 ppm) in November 2020. Nearby blocks of *C. sinensis* that were not treated with GA were used as negative controls. Trees were investigated in June 2021. (a) Trunk diameter measured at ~20 cm above the ground. (b) Tree height above the ground from the soil surface to the apical point of the plant. (c) Canopy volume of the GA treated and untreated control trees. Data shown are means of 10 replicated trees (n = 10). Statistical analysis was performed using two-tailed Student's t-test and a significant difference between GA treated and untreated control trees was determined at $P < 0.05$. Box plots: central line represents the median, box edges delimit lower and upper quartiles, whiskers display the highest and lowest data points, and dots above the boxes are outliers. Source data are provided as a Source Data file.

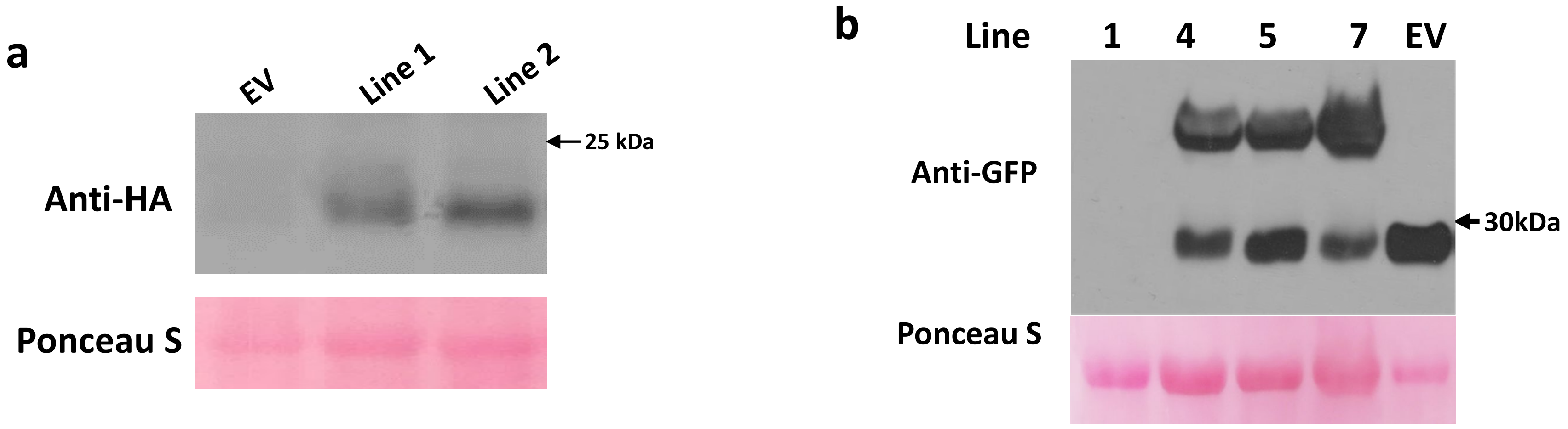

Fig. S15. Confirmation of transgenic plants using immunoblotting. a. The transgenic 'Pineapple' sweet orange (*Citrus sinensis* (L.) Osbeck) plants were confirmed using immunoblotting using the anti-HA antibody. Ponceau S stained membrane was used as a loading control. A citrus plant transformed with empty vector was used as the negative control. b. Transgenic Arabidopsis constitutively expressing CLas proteins was validated by immunoblotting. Immunoblotting was conducted using the anti-GFP antibody. Ponceau S stained membrane was used as a loading control. Empty vector transformed Arabidopsis was used as the negative control.
