# Supplementary material for: Citrus Huanglongbing is a pathogen-triggered immune disease that can be mitigated with antioxidants and gibberellin
Source: Nat Commun. 2022 Jan 26;13:529. doi: 10.1038/s41467-022-28189-9 (PMC8791970; doi:10.1038/s41467-022-28189-9)
Supplement: Supplementary file 3 — Description of Additional Supplementary Files [file 41467_2022_28189_MOESM3_ESM.pdf]

Title: Supplementary Data 1:
Description: Differentially expressed genes between GA and non-GA (nGA) treated Citrus sinensis protoplast cells in the presence of 1.8 mM H_2O_2.
