# Supplementary material for: Citrus Huanglongbing is a pathogen-triggered immune disease that can be mitigated with antioxidants and gibberellin
Source: Nat Commun. 2022 Jan 26;13:529. doi: 10.1038/s41467-022-28189-9 (PMC8791970; doi:10.1038/s41467-022-28189-9)
Supplement: Supplementary file 5 — Reporting Summary [file 41467_2022_28189_MOESM5_ESM.pdf]

nature portfolio

Corresponding author(s): Nian Wang

Last updated by author(s): Dec 10, 2021

Reporting Summary

Nature Portfolio wishes to improve the reproducibility of the work that we publish. This form provides structure for consistency and transparency in reporting. For further information on Nature Portfolio policies, see our Editorial Policies and the Editorial Policy Checklist.

Statistics

For all statistical analyses, confirm that the following items are present in the figure legend, table legend, main text, or Methods section.

n/a	Confirmed	
☐	☒	The exact sample size (*n*) for each experimental group/condition, given as a discrete number and unit of measurement
☐	☒	A statement on whether measurements were taken from distinct samples or whether the same sample was measured repeatedly
☐	☒	The statistical test(s) used AND whether they are one- or two-sided<br>*Only common tests should be described solely by name; describe more complex techniques in the Methods section.*
☒	☐	A description of all covariates tested
☐	☒	A description of any assumptions or corrections, such as tests of normality and adjustment for multiple comparisons
☐	☒	A full description of the statistical parameters including central tendency (e.g. means) or other basic estimates (e.g. regression coefficient) AND variation (e.g. standard deviation) or associated estimates of uncertainty (e.g. confidence intervals)
☐	☒	For null hypothesis testing, the test statistic (e.g. *F*, *t*, *r*) with confidence intervals, effect sizes, degrees of freedom and *P* value noted<br>*Give P values as exact values whenever suitable.*
☒	☐	For Bayesian analysis, information on the choice of priors and Markov chain Monte Carlo settings
☒	☐	For hierarchical and complex designs, identification of the appropriate level for tests and full reporting of outcomes
☒	☐	Estimates of effect sizes (e.g. Cohen's *d*, Pearson's *r*), indicating how they were calculated

Our web collection on statistics for biologists contains articles on many of the points above.

Software and code

Policy information about availability of computer code

Data collection	We do not use any software to collect the data.
Data analysis	SAS statistical software V9.4, HISAT2 V2.2.1, SAMtools V1.2, HTSeq-count V0.11.2, DESeq2 V1.30.1, Pheatmap V1.0.12, Limma V3.46.0, R V4.0.5, gplots V3.1.1, agriGO v2.0.

For manuscripts utilizing custom algorithms or software that are central to the research but not yet described in published literature, software must be made available to editors and reviewers. We strongly encourage code deposition in a community repository (e.g. GitHub). See the Nature Portfolio guidelines for submitting code & software for further information.

Data

Policy information about availability of data

All manuscripts must include a data availability statement. This statement should provide the following information, where applicable:

- Accession codes, unique identifiers, or web links for publicly available datasets
- A description of any restrictions on data availability
- For clinical datasets or third party data, please ensure that the statement adheres to our policy

The raw RNA-seq data generated in this study were deposited in the NCBI Bioproject database under the accession number PRJNA780217. The public transcriptomic data were generated from NCBI GEO and SRA databases under accession numbers GSE33003, GSE33004, GSE33459, GSE29633, GSE33373, GSE101381, and SRP022979.

Field-specific reporting

Please select the one below that is the best fit for your research. If you are not sure, read the appropriate sections before making your selection.

☒ Life sciences ☐ Behavioural & social sciences ☐ Ecological, evolutionary & environmental sciences

For a reference copy of the document with all sections, see nature.com/documents/nr-reporting-summary-flat.pdf

Life sciences study design

All studies must disclose on these points even when the disclosure is negative.

Sample size	No statistical methods were used to predetermine sample size. Sample size for protection assays was chosen based on previous experience or according to the experiments and previous publications on similar methods. H2O2 concentrations were quantified following the procedure described elsewhere (Kumar et al. 2011). qRT-PCR was conducted following the method described by Pang et al. (2020 Plant Physiology, 184 Pages 792–805). Ion leakage was conducted following the method described by Teper et al. 2020 (PLoS pathogens 16 (9), e1008886). Starch was quantified following the method described by Ribeiro et al. 2021 (Plant Molecular Biology 106, 349–366). Foliar spray and trunk injection were conducted following the method described by Li et al. 2021 (Phytopathology 111, 1095-1103).
Data exclusions	No
Replication	To ensure the reliability of experimental results, we had at least three biologic replicates for each experiment, which have been described in the relevant figure legends.
Randomization	For field trials, the experiment was a completely randomized design with 5 treatments. Each treatment consisted of four trees.
Blinding	Samples in each experiment were harvested as well as processed by multiple researchers. Experiments were independently validated or performed by different researchers to ensure reproducibility of the data.

Reporting for specific materials, systems and methods

We require information from authors about some types of materials, experimental systems and methods used in many studies. Here, indicate whether each material, system or method listed is relevant to your study. If you are not sure if a list item applies to your research, read the appropriate section before selecting a response.

Materials & experimental systems

n/a	Involved in the study	
☐	☒	Antibodies
☒	☐	Eukaryotic cell lines
☒	☐	Palaeontology and archaeology
☒	☐	Animals and other organisms
☒	☐	Human research participants
☒	☐	Clinical data
☒	☐	Dual use research of concern

Methods

n/a	Involved in the study	
☒	☐	ChIP-seq
☒	☐	Flow cytometry
☒	☐	MRI-based neuroimaging

Antibodies

Antibodies used	HA Tag Antibodies (Roche, 11867423001); GFP antibodies (Sigma-Aldrich, SAB4301138)
Validation	HA and GFP antibodies were validated by immunoblot of target proteins present in transgenic plants and absence in the wild-type plants. We have western plot figures present in the supplementary figures (fig. S15).
